# Introgression of *Sub1 (SUB1) QTL* in mega rice cultivars increases ethylene production to the detriment of grain- filling under stagnant flooding

**DOI:** 10.1038/s41598-019-54908-2

**Published:** 2019-12-06

**Authors:** Sandhya Rani Kuanar, Kutubuddin Ali Molla, Krishnendu Chattopadhyay, Ramani Kumar Sarkar, Pravat Kumar Mohapatra

**Affiliations:** 1 ICAR-National Rice Research Institute, Cuttack, Odisha 753006 India; 2 School of Life Science, Sambalpur University, Jyoti vihar, Sambalpur, 768019 India; 3 Present Address: Anchal College, Padampur, 768036 India

**Keywords:** Plant sciences, Plant physiology

## Abstract

In the recent time, *Submergence1* (*Sub1)QTL*, responsible for imparting tolerance to flash flooding, has been introduced in many rice cultivars, but resilience of the QTL to stagnant flooding (SF) is not known. The response of *Sub1*-introgression has been tested on physiology, molecular biology and yield of two popular rice cultivars (Swarna and Savitri) by comparison of the parental and *Sub1-*introgression lines (SwarnaSub1 and SavitriSub1) under SF. Compared to control condition SF reduced grain yield and tiller number and increased plant height and *Sub1*- introgression mostly matched these effects. SF increased ethylene production by over-expression of ACC-synthase and ACC-oxidase enzyme genes of panicle before anthesis in the parental lines. Expression of the genes changed with *Sub1-*introgression, where some enzyme isoform genes over-expressed after anthesis under SF. Activities of endosperm starch synthesizing enzymes SUS and AGPase declined concomitantly with rise ethylene production in the *Sub1*-introgressed lines resulting in low starch synthesis and accumulation of soluble carbohydrates in the developing spikelets. In conclusion, *Sub1-*introgression into the cultivars increased susceptibility to SF. Subjected to SF, the QTL promoted genesis of ethylene in the panicle at anthesis to the detriment of grain yield, while compromising with morphological features like tiller production and stem elongation.

## Introduction

The uncertainty of occurrence, duration, and amount of rainfall affect productivity of the rainfed lowland and flood-prone rice ecosystem^1–3^. During wet cultivation more than 7 million hectares of rice agro-ecosystem in eastern India are affected by medium depth (25–50 cm) to deep water (50–100 cm) stagnant flooding (SF)^4^. Thus, acceptance of modern rice cultivars with no security for flooding, stagnant or flash, is not a viable option in flood-prone ecosystem. Modern rice cultivars need stability of growing conditions and compromise on yield subjected to floods. As of today, rice gene bank is short of appropriate high yielding variety, which can tolerate diverse flooding conditions. Recently, *Submergence-1* (*Sub1*) *QTL*, which imparts tolerance to flash flooding, has been introgressed in some rice cultivars^3,5,6^. The modified genotypes have shown greater tolerance under flash flooding^7,8^. Presence of *Sub1* reduces pace of reserved carbohydrate consumption during submergence and conserve assimilates for recovery of growth and survival. The *Sub1* locus contains three genes, *viz. Sub1A*, *Sub1B* and *Sub1C*. *Sub1A* is present in limited *indica* rice cultivars, whereas *Sub1B* and *Sub1C* are omni-present in *japonica* and *indica* cultivars^9^. *Sub1A* gene encoding ERF transcription factor confers tolerance to flooding in rice^8^. It reduces ethylene sensitivity of rice plants under submergence and thereby checks rapid stem elongation and chlorophyll degradation^9,10^. It is conjectured that mega rice cultivars with *Sub1 QTL* introgressed in them, can also confer resistance to SF similar to flash flooding, but information on various functional aspects of grain filling is scant^11,12^.

Capacity of spikelets for grain-filling is undermined by evolution of ethylene at anthesis^13,14^. Ethylene production rate is negatively correlated with grain-filling rate^15^ and assimilates not used in grain growth accumulate in under-developed spikelets^16,17^. Typically, a high level ethylene production at anthesis reduces grain-filling of spikelets by slackening activities of starch synthesizing enzymes, such as, sucrose synthase (SUS), ADPglucose pyrophosphorylase (AGPase) and starch synthase (SS)^15,18–21^. Ethylene evolution down-regulates expression of the enzyme genes in the inferior spikelets of panicle^22^.The role of the hormone in grain-filling also becomes crucial during stress^23,24^. However, information is scant as to how ethylene regulates activities of the enzymes when plants are subjected to SF. Yang *et al*.^25^ recently discussed the signal cascade leading to repression or activation of multitude of ethylene response genes in *Arabidopsis* and rice. Ethylene perception by receptors in endoplasmic reticulum inactivates the receptors and the Raf-like ser/thr protein kinase CTR1: inactive CTR1 fails to phosphorylate EIN2, a membrane bound protein and primary ethylene signal transducer down stream, leading to cleavage of its C-terminus. The split EIN2 C-terminus enters into the nucleus and activates master transcription factors EIN3 and ELI1, which subsequently stimulate expression of *ERF1* and several ethylene responsive genes. Bailey-Serres *et al*.^26^ proposed that *Sub1A* expression is induced by ethylene production during submergence, which in turn activates *ERF* expression. The ERF transcription factor slackens ethylene production and gibberellin responsiveness that conserve carbohydrate reserve otherwise used for stem elongation under flooding^27^. Based on a simple logic that expression of *Sub1 QTL* encoding ERF in the *Sub1*-introgressed rice cultivars under SF can underscore the expression of ethylene response genes and thereby ameliorate adverse effects on grain-filling, an experiment has been designed to evaluate two popular rice cultivars and their *Sub1*-introgressed near isogenic lines to different levels of SF in simulated conditions.

## Results

### Yield and yield attributes

On an average dry matter production, yield and yield attributing parameters such as panicle weight, grain weight per panicle, filled grain(%), panicle number m^–2^, straw weight m^–2^ and grain weight m^–2^ had greater values in 2014 compared to 2013 (Tables S1, S2). In both the years the response of the cultivars to SF was almost similar. SF decreased various yield and yield attributing parameters in all cultivars, but the effect was higher in cultivars with *Sub1*. The damage was greater in SwarnaSub1 compared to SavitriSub1. The reduction in grain weight m^−2^ under SF for Swarna in 2013 and 2014 were 26.9 and 27.4% respectively, while the corresponding figures for SwarnaSub1 were 70.1 and 60.1% respectively. Similarly SF reduced grain weight per m^−2^ in SavitriSub1 by 47.1 and 50.5% in 2013 and 2014 respectively, as compared to corresponding figures of 32.6 and 31.1% in Savitri. The *Sub1-*introgression also depressed shoot biomass and other grain yield parameters like panicle grain weight, panicle number m^−2^ and filled grain% significantly (p** < *0.01*) of both cultivars. Flowering and maturity dates were delayed under SF compared to control condition irrespective of the cultivar variation (Table S2). The delay was longer for cultivars with *Sub1* compared to their respective parents without *Sub1*. SF decreased panicle grain weight and filled grain(%) of the main shoot consistently in all cultivars, although the effect was not always significant. In general, introgression of *Sub1* depressed these parameters significantly (p* < *0.05*) under SF.

### Plant height and tiller number

**Figure 1:**
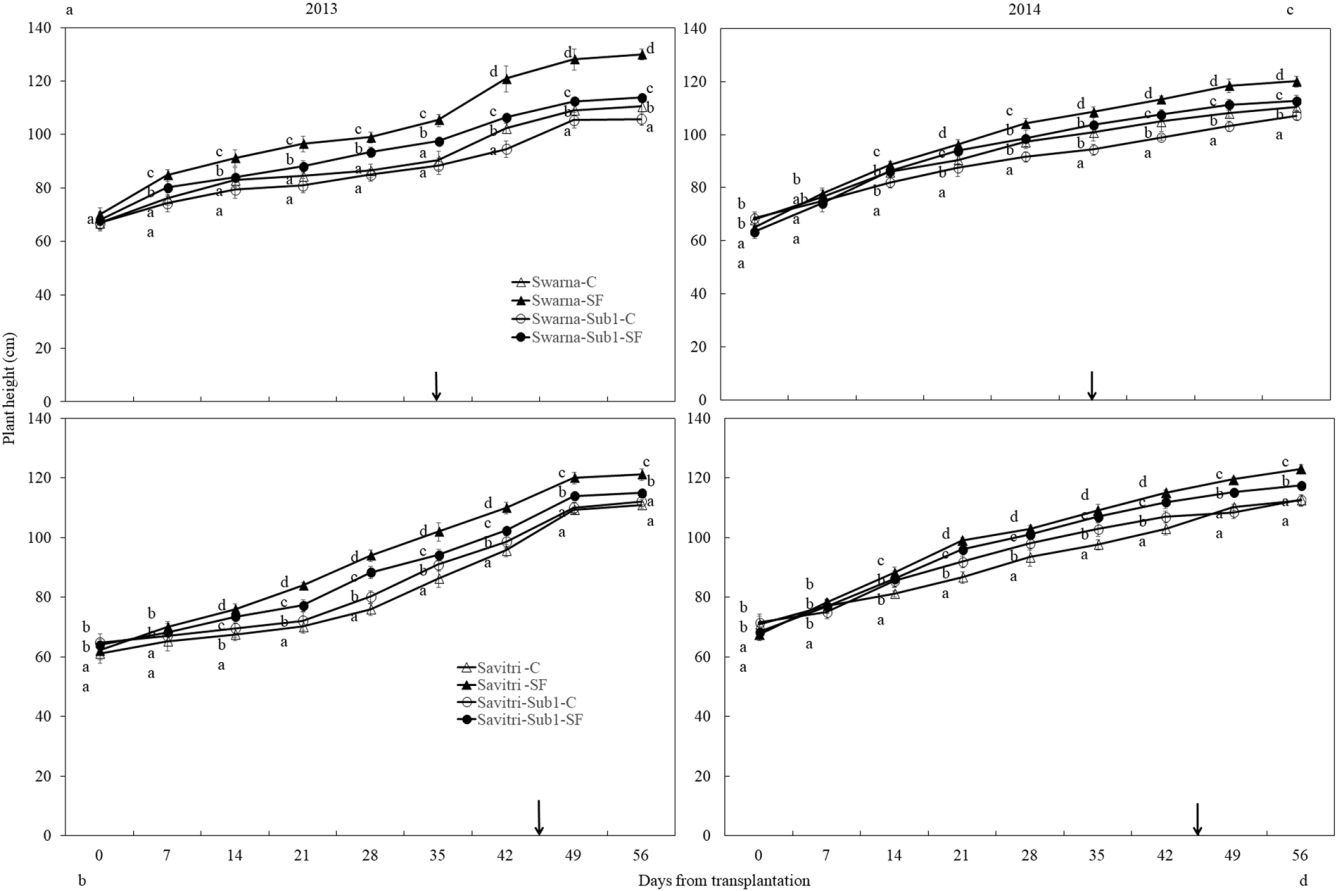
Effect of SF on height of rice cultivars Swarna and Savitri with and without *Sub1* in 2013 (**a,b**) and 2014 (**c,d)**. The vertical bars indicate mean ± standard deviation (n = 9) at p* < *0.05*. Arrow- Anthesis. Different small case letter at a specific measuring time signifies significant statistical difference here and in other figures as well.

**Figure 2:**
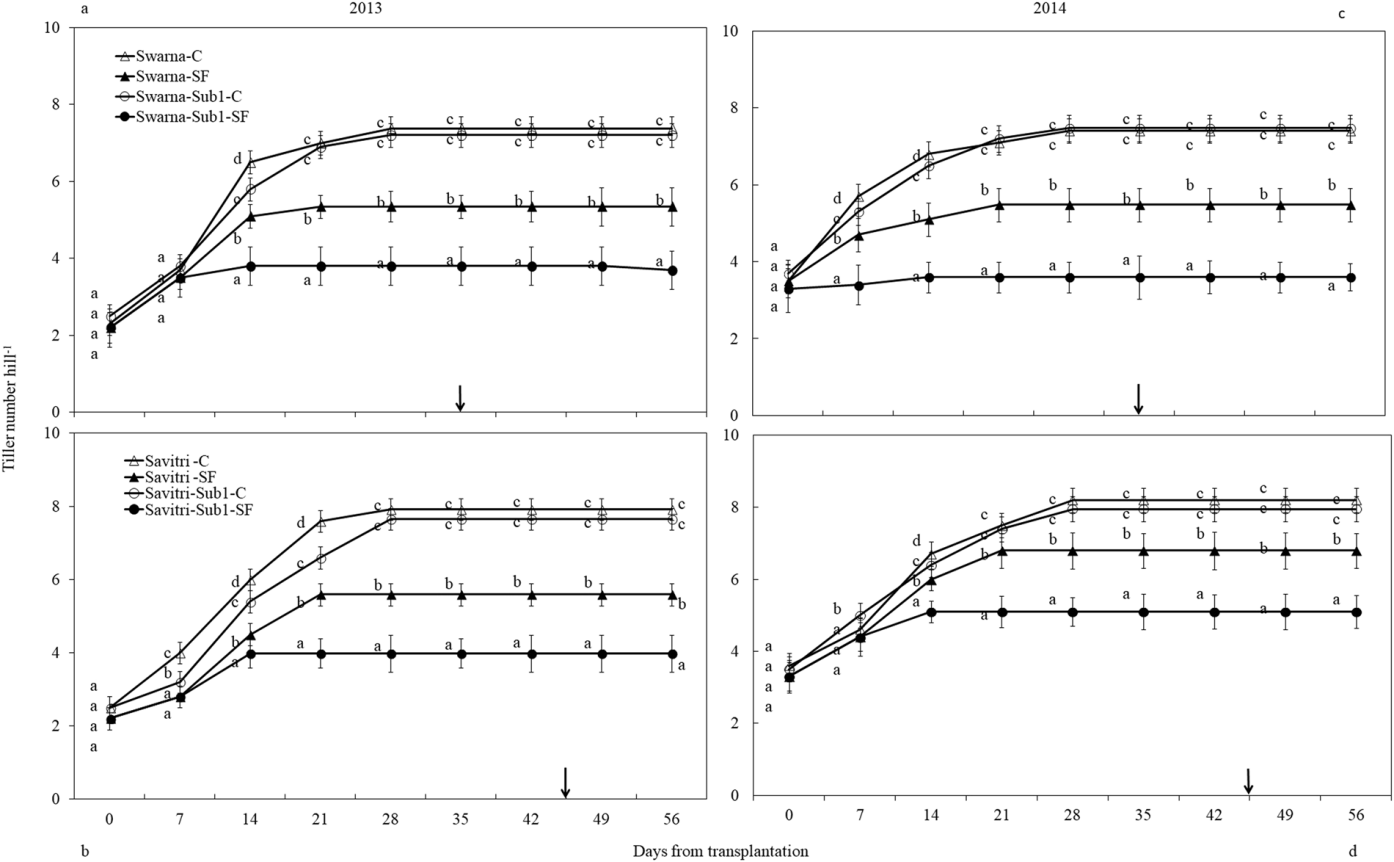
Effect of SF on tiller number of rice cultivars Swarna and Savitri with and without *Sub1* in 2013 (**a,b**) and 2014 (**c,d**). The vertical bars indicate mean ± standard deviation (n = 9) at p* < *0.05*.

Plant height was identical for all cultivars at transplantation and increased temporally until day 56. Compared to 2013 plant height was smaller in 2014. Introgression of *Sub1* did not change the pattern significantly in the control (Fig. 1). SF increased plant height of all cultivars consistently, but the elongation growth was greater in cultivars without *Sub1* compared to cultivars with *Sub1* (p** < *0.01*). Before the imposition of SF, tiller number per hill was almost similar in cultivars with and without *Sub1* (Fig. 2). Savitri had more tillers compared to Swarna. In Swarna the number increased during the first two weeks after transplanting and remained stable thereafter. In SwarnaSub1 tiller initiation continued for a week more, but it did not contribute to increase of number compared to its un-modified counterpart. The response of Savitri in tillering to the genotypic modification and SF was similar to Swarna. Tiller initiation in Savitri continued until the third week of transplantation and for a week more in the modified genotype although there was no difference in final tiller numbers. Imposition of SF reduced tillering in Savitri and SavitriSub1, but the impact was significantly greater on the latter (p** < *0.01*) (Fig. 2).

### Grain weight and growth

Average grain weight of panicle of main shoot increased slowly in a sigmoidal pattern during post-anthesis period in all cultivars (Fig. S1). The temporal increase of grain weight was greater in control compared to SF. Variation in cultivar, flooding treatment and days after anthesis significantly (p** < *0.01*) influenced the grain weight gain in both the years. Between the cultivars Savitri had larger grain weight compared to Swarna. SF impacted grain weight significantly during the post-anthesis period and extended the period of maturation in all cultivars. Introgression of the *Sub1* in Swarna reduced grain weight gain, but it was not as much effective for Savitri. SavitriSub1 possessed better grain growth with passage of time as against its parent Savitri.

Rate of grain-filling in the panicle of the main shoot exhibited a curvilinear pattern temporally in the post-anthesis period in all cultivars (Fig. S2). The rate increased up to day 13 after anthesis and declined thereafter till maturity in plants under control condition. In comparison, under SF, grain-filling rate increased slowly to day 16 and declined continuously thereafter to day 28. In the F-test, cultivar, treatment and days after anthesis and their interaction significantly (***p < *0.001*) influenced the grain-filling rate, but impact of the SF treatment was wider than the others. In control condition grain-filling rate was almost similar in Swarna and SwarnaSub1. Under SF, however, grain-filling rate was greater most of the time in Swarna compared to SwarnaSub1. The trend was somewhat different between Savitri and SavitriSub1. The grain-filling rate was greater in SavitriSub1 compared to Savitri both in control and SF conditions mostly at the middle stage of grain growth. Grain growth rate ceased at least 3 days early in control compared to SF condition.

### Sugar and starch concentration of developing grain/spikelet

**Figure 3:**
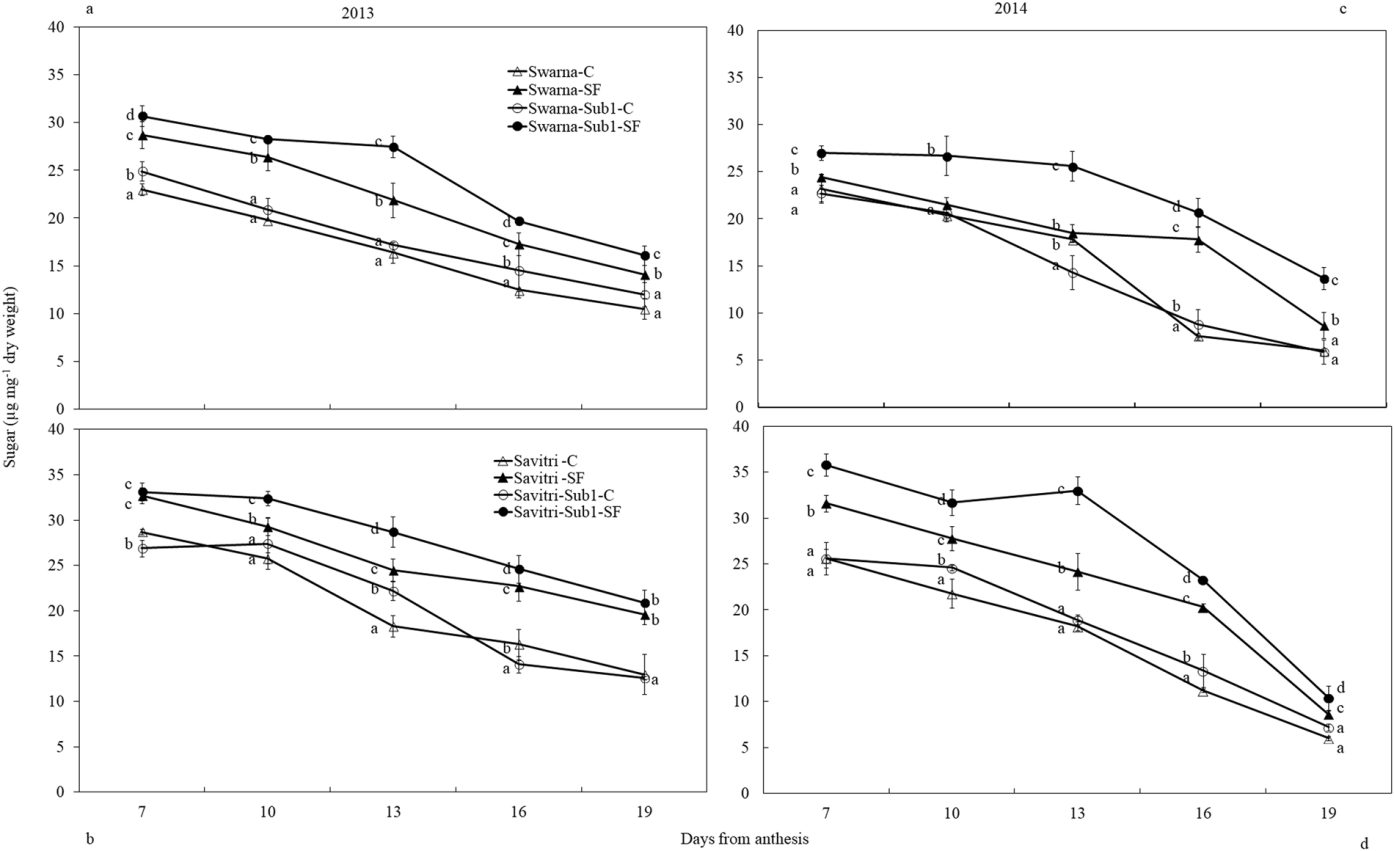
Effect of SF on soluble sugar concentration of main panicle of rice cultivars Swarna and Savitri with and without *Sub1* in 2013 (**a,b**) and 2014 (**c,d**). The vertical bars indicate mean ± standard deviation (n = 3) at p* < *0.05*.

**Figure 4:**
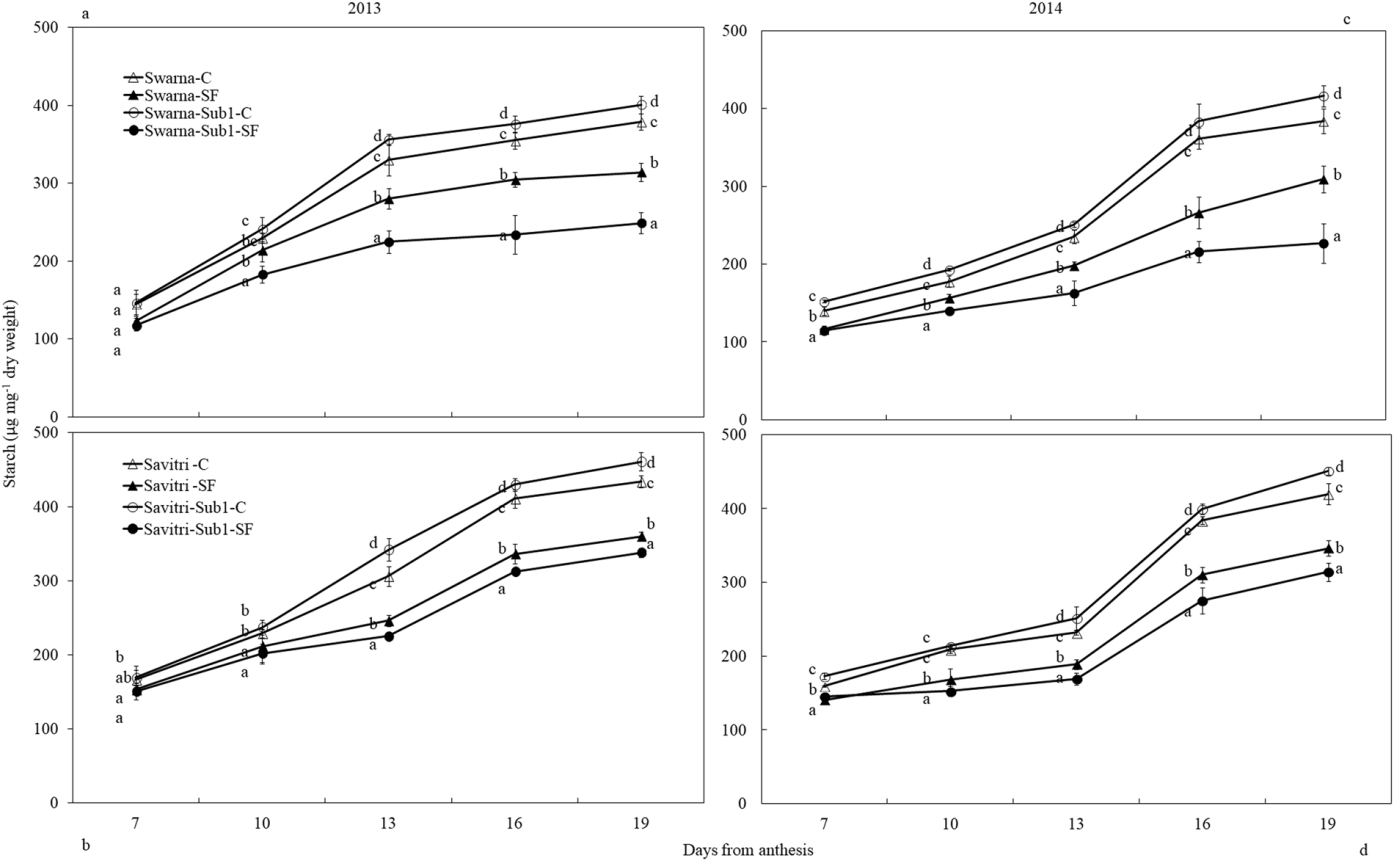
Effect of SF on starch concentration of main panicle of rice cultivars Swarna and Savitri with and without *Sub1* in 2013 (**a,b**) and 2014 (**a,d**). The vertical bars indicate mean ± standard deviation (n = 3) at p* < *0.05*.

Soluble sugar and starch concentrations of developing grains of panicle on main shoot were measured between days 7 and 19 post-anthesis (Figs. 3, 4). In the F-test, cultivar, SF-treatment and days after anthesis significantly (p*** < *0.001*) influenced the soluble sugar concentration. The concentration declined progressively with passage of time in all cultivars. Imposition of SF increased the level on all sampling occasions (Fig. 3). Between the cultivars with and without *Sub1*, the concentration was higher in the former compared to the latter. This effect was consistent on all sampling occasions for Swarna, but narrowed down to a low level towards the end period of sampling for Savitri.

In contrast to soluble sugar, starch concentration in developing grain increased gradually with time between days 7 and 19 post-anthesis and the concentration was higher in control compared to SF (Fig. 4). Imposition of SF reduced starch accumulation in the developing grains. In the F-test, cultivar, treatment and days after anthesis significantly (p*** < *0.001*) influenced the starch concentration in developing grains. Under control condition, starch concentration in developing grains was occasionally greater in cultivars with *Sub1* compared to their respective parents lacking *Sub1*. Contrastingly, under SF, cultivars with *Sub1* exhibited less accumulation of starch during the period of grain development in comparison with their counterpart without the *QTL*. Under SF, the difference between the starch content between Swarna and SwarnaSub1 (**p < *0.01*) was greater than the difference between Savitri and SavitriSub1 (Fig. 4).

### Sucrose synthase (SUS) and ADPglucose pyrophosphorylase (AGPase) activities

**Figure 5:**
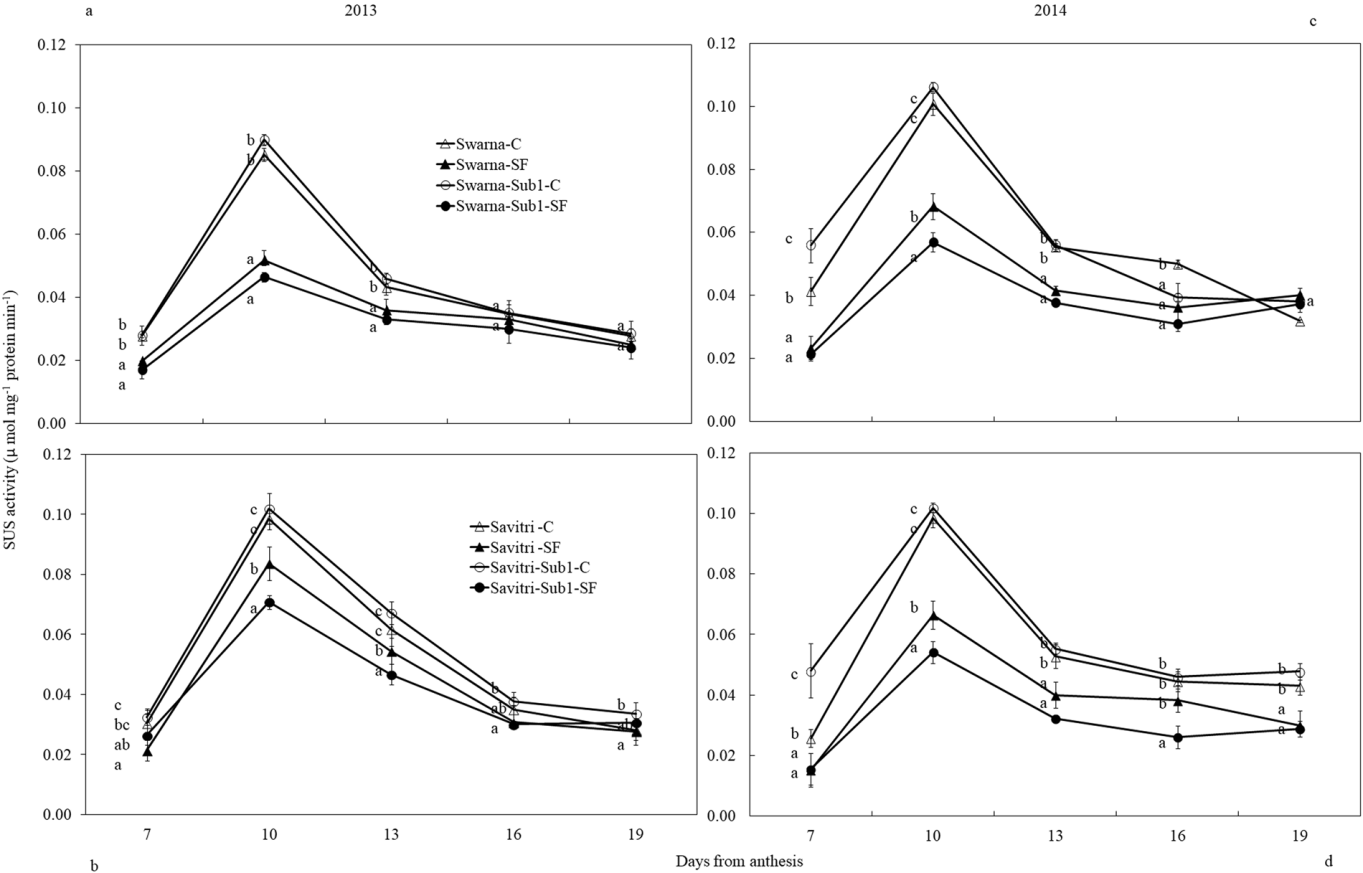
Effect of SF on activity of sucrose synthase of spikelets of main panicle in rice cultivars Swarna and Savitri with and without *Sub1* in 2013 (**a,b**) and 2014 (**c,d**). The vertical bars indicate mean ± standard deviation (n = 3) at p* < *0.05*.

SUS activity of the developing grains increased between days 7 and 10 post-anthesis and declined thereafter to a minimum level in the cultivars both under control and SF conditions (Fig. 5). Except occasional differences, SUS activity did not vary much between the cultivars with and without *Sub1* under control condition. However, SF diminished SUS activity significantly (***p < *0.001*) in all the cultivars compared to control condition. Under SF cultivars with *Sub1* had low SUS activity compared to control conditions on 10, 13 and 16 days of anthesis.

**Figure 6:**
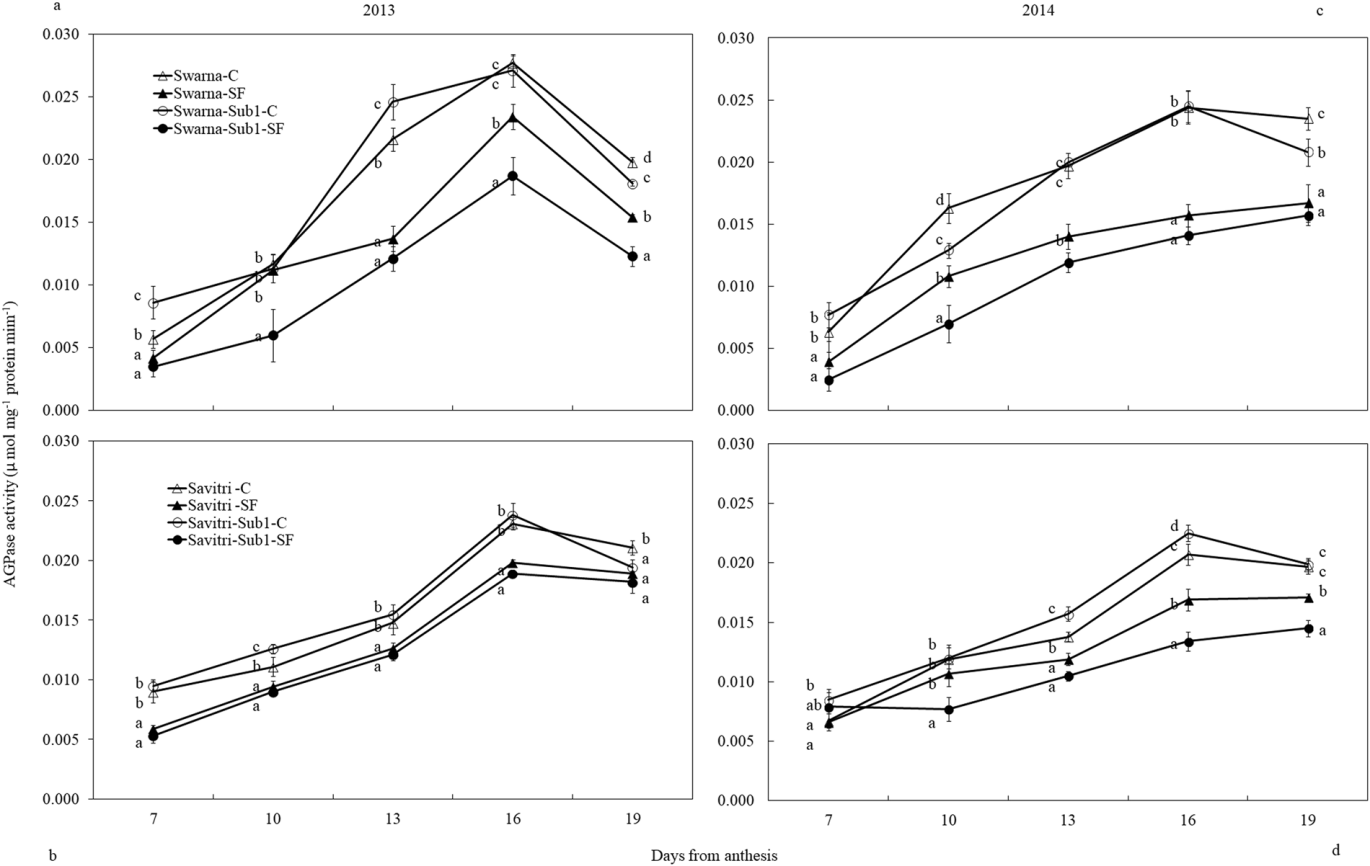
Effect of SF on activity of ADP-glucose pyrophosphorylase of spikelets of main panicle in rice cultivars Swarna and Savirti with and without *Sub1* in 2013 (**a,b**) and 2014 (**c,d**). The vertical bars indicate mean ± standard deviation (n = 3) at p* < *0.05*.

AGPase activity the developing grains of the panicle of main shoot showed pattern of increase different from that of SUS activity in the post-anthesis period (Fig. 6). AGPase activity increased gradually up to day 16 post-anthesis both under control and SF in all cultivars and changed marginally thereafter with the exception of year 2013, in which the activity declined on day 19. Difference in treatment, cultivar and days after anthesis significantly (**p < *0.01*) influenced AGPase activity of the grains in the F-test. Compared to Savitri, AGPase activity was higher in Swarna genotypes. Similar to SUS, AGPase activity was always low under SF compared to control. Under control condition the activity of AGPase was almost similar between the cultivars with and without *Sub1*. However, imposition of SF greatly altered the pattern of activity. Cultivars with *Sub1* showed less AGPase activity under SF compared to control.

### Ethylene evolution

**Figure 7:**
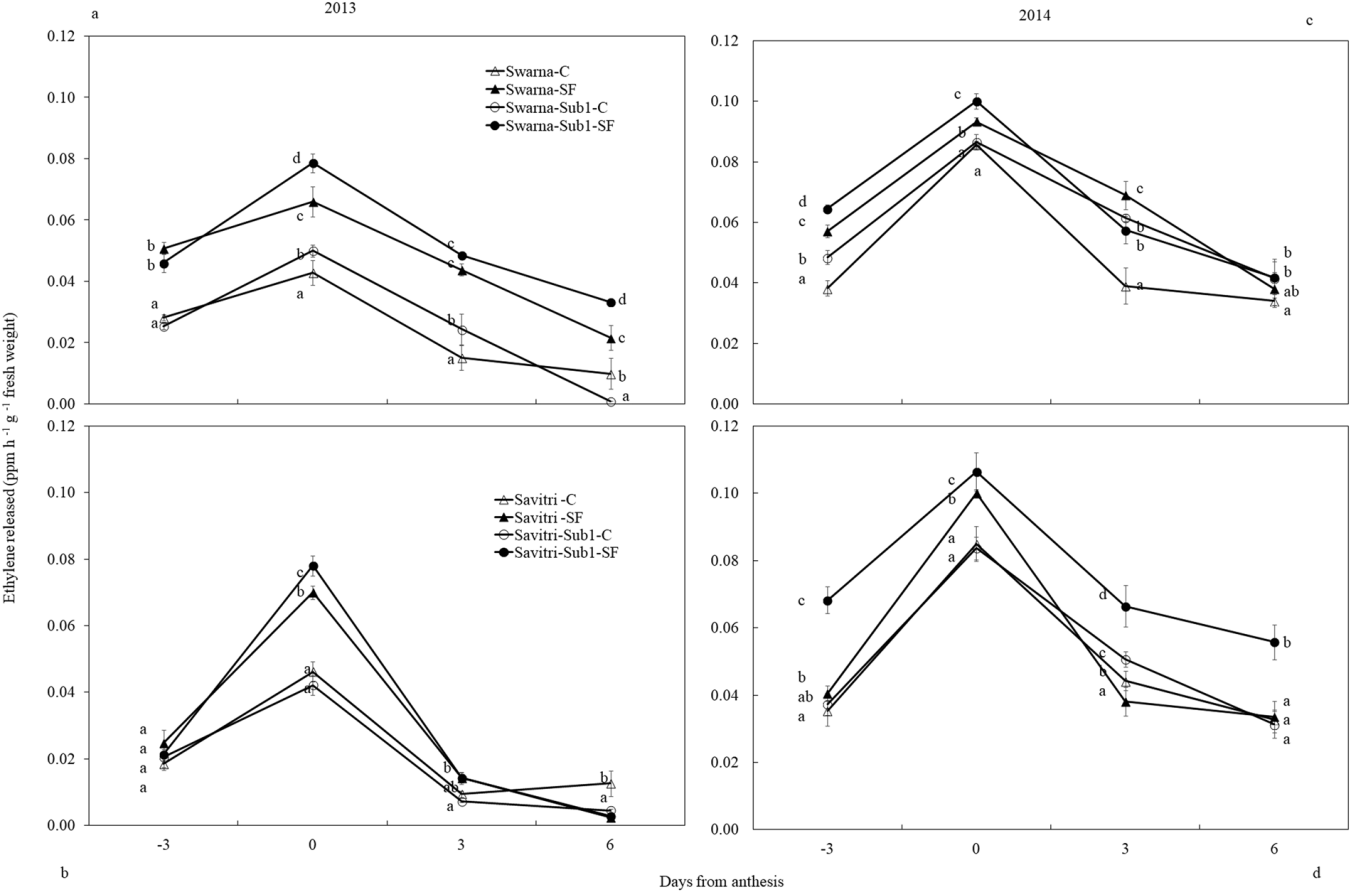
Effect of SF on ethylene concentration of main panicle in rice cultivars Swarna and Savitri with and without *Sub1* in 2013 (**a,b**) and 2014 (**c,d**). The vertical bars indicate mean ± standard deviation (n = 3) at p* < *0.0.5*.

Evolution of ethylene from the panicle of the main shoot increased from three days before anthesis and culminated to a peak at anthesis irrespective of cultivar variation (Fig. 7). Subsequently it declined rapidly in the next six days to a minimum level. In the F-test, cultivar, flooding treatment and days after anthesis significantly (p*** < *0.001*) influenced the ethylene release from the panicle in both the years. Comparison of two years data revealed that under control condition ethylene production did not change much between cultivars with and without *Sub1*. In 2013, ethylene release started to decline very fast from anthesis, and within next 6 days ethylene production reached almost to a ground level under control condition. In 2014, the result was somewhat different as noticeable amount of ethylene was found to be released at 6 days post-anthesis. The release of ethylene was more pronounced in cultivars with *Sub1* compared to the cultivars without *Sub1*.

### Quantitative real time polymerase chain reaction (qRTPCR) for ACC synthase and ACC oxidase gene expression

Under SF, all the 12 paralogs of ethylene producing enzymes (ACC synthase and ACC oxidase) were found to be up-regulated three days before anthesis in Swarna and Savitri (Table 1). In comparison, expression of the paralogs mostly reversed in cultivars with *Sub1*; the expression of all paralogs except *OsACO*3 in SwarnaSub1 and *OsACO4* in SavitriSub1 was down regulated at this stage. With passage of time expression of the paralogs changed in cultivars without *Sub1* as most of the genes showed a down-regulation trend. The changes were more discernible in Swarna compared to Savitri. At anthesis, expression of paralogs *OsACS3* and *OsACO3* only was up-regulated by SF in Swarna, whereas in Savitri, six paralogs, viz., *OsACS1*, *OsACS2*, *OsACS3*, *OsACS5*, *OsACS6* and *OsACO5* over-expressed. The effect of SF on expression of the papralogs was not exactly identical in cultivars with *Sub1* at anthesis, because one (*OsACS5)* of Swarna-Sub1 and eight (*OsACS1, OsACS2, OsACS5, OsACS6, OsACO1, OsACO2, OsACO4, OsACO5*) of SavitriSub1 paralogs over-expressed, but not the others. At three days post-anthesis, *OsACS5, OsACO3*, *OsACO5* and *OsACO7* showed over expression in Swarna, while in Swarna-Sub1, only *OsACO5* and *OsACO3* over-expressed under SF. The expression pattern of the paralogs in Savitri was not identical to Swarna in response to SF at this stage. Compared to the down-regulation trend in expression of most of the genes in Swarna, many paralogs of the enzyme genes over-expressed in Savitri on this occasion. All 12 paralogs expressed up- and down-regulation identically in both Savitri and SavitriSub1 under SF. Comparison of magnitude of expression of various paralogs between Swarna with and without *Sub1* subjected to SF revealed differential expression of the genes between the two genotypes. Most of the paralogs in Swarna over-expressed prior to anthesis, which diminished with passage of time (Fig. S3). In contrast, most of the paralogs under expressed with rare instances of over expression prior to anthesis in SwarnaSub1; the under-expression continued to increase with passage of time up to day 3 post-anthesis. Similarly, expression of many paralogs was up regulated in Savitri before anthesis, which declined considerably at early post-anthesis stage. In SavitriSub1, however, most of the paralogs under-expressed prominently before anthesis, but the level of expression recuperated to ground level at anthesis and post-anthesis stages.

**Table 1:** Relative gene expression of various isoforms of ACC synthase (OsACS) and ACC oxidase (OsACO) enzymes in the developing spikelets of panicle of the main shoot of rice cultivars Swarna and Savitri with and without *Sub1*, under SF compared to control.

Cultivars	Crop Growth stage	1	2	3	4	5	6	7	8	9	10	11	12
Swarna	3 d before anthesis	**↑**	**↑**	**↑**	**↑**	**↑**	**↑**	**↑**	**↑**	**↑**	**↑**	**↑**	**↑**
Swarna-Sub1	3 day before anthesis	↓	↓	↓	↓	↓	↓	↓	↓	↑	↓	↓	↓
Swarna	Anthesis	↓	↓	↑	↓	↓	↓	↓	↓	↑	↓	↓	↓
Swarna-Sub1	Anthesis	↓	↓	↓	↓	↑	↓	↓	↓	↓	↓	↓	↓
Swarna	3 day after anthesis	↓	↓	↓	↓	↑	↓	↓	↓	↑	↓	↑	↑
Swarna-Sub1	3 day after anthesis	↓	↓	↓	↓	↑	↓	↓	↓	↑	↓	↓	↓
Savitri	3 day before anthesis	**↑**	**↑**	**↑**	**↑**	**↑**	**↑**	**↑**	**↑**	**↑**	**↑**	**↑**	**↑**
Savitri-Sub1	3 day before anthesis	↓	↓	↓	↓	↓	↓	↓	↓	↓	↑	↓	↓
Savitri	Anthesis	↑	↑	↑	↓	↑	↑	↓	↓	↓	↓	↑	↓
Savitri-Sub1	Anthesis	↑	↑	↓	↓	↑	↑	↑	↑	↓	↑	↑	↓
Savitri	3 day after anthesis	↑	↓	↑	↓	↑	↓	↑	↓	↑	↑	↓	↑
Savitri-Sub1	3 day after anthesis	↑	↓	↑	↓	↑	↓	↑	↓	↑	↑	↓	↑

↑,Up-Regulation; ↓, Down-Regulation; [1- OsACS1, 2-OsACS2, 3-OsACS3, 4 -OsACS4, 5-OsACS5, 6-OsACS6, 7-OsACO1, 8- OsACO2, 9-OsACO3, 10-OsACO4, 11-OsACO5, 12-OsACO7].

## Discussion

Deep water rice and some lowland cultivars survive SF, although many cultivars die within 7 days^26^ of stress. Deep water rice survives drowning through matchable growth response by promoting internode elongation and keeps afloat the upper leaves above water level for normal gas exchange. Hattori *et al*.^28^ reported that two ERF DNA binding proteins SNORKEL1 and SNORKEL2 allow the plant to adapt to deep water, where ethylene generated in plant organs under submergence down-regulates ABA response, thereby permitting GA action for stem elongation^27^. Conversely, the mechanism of flood tolerance in lowland rice is different. Another ERF located at the *Sub1* polygenic locus^8^, restrains increase of plant height and conserve reserve carbohydrates otherwise used in stem elongation for development of new leaves upon de-submergence. Out of the three *Sub1*genes, *Sub1A* in particular slackens ethylene production restricting GA responsiveness for stem elongation^27^. As proposed, in both deep water and lowland rice, ethylene production in the stem is the key factor controlling resilience to flooding, although the mechanism of one is opposite to the other. Conceptually, the present study does not conflict with the role of ethylene either in antithetical growth responses of rice under SF or induction of the robust *QTL Sub1* that counters ethylene induced stem elongation. It embodies a comprehensive account of SF-induced changes in ethylene production and its consequential effects on grain-filling and other phenotypic attributes of two lowland cultivars, and ineffectiveness of the *Sub1-* introgression for resilience to SF.

The cultivars with and without *Sub1* gave equal biomass and grain yield under normal condition but not under SF (Tables S1, S2). The *Sub1-*introgressed lines became quiescent and showed less elongation growth under SF. Generally cultivars with *Sub1* survive complete submergence through restriction of stem elongation^26,29–31^ and conserve non-structural carbohydrates for quick regeneration^31,32^. Our finding is in conformity with the ERF of *Sub1A* locus working as deterrent for GA-promoted stem elongation under submergence^8,29^ (Fig. 1). Elongation growth by the Sub1-ERF is useful for survival under complete submergence, but not for partial SF where the exposed parts perform photosynthesis. Thus, the semi-dwarf genotypes used showed better growth compared to the *Sub1-*introgressed lines (Table S1). The semi-dwarf rice with *sd1-allele* is gibberellin-deficient for stem elongation^33,34^. In our study plant height increased under SF (Fig. 1), because ethylene generated under submergence^35^ promoted gibberellin synthesis. They acclimated to the stress and compensated growth by delaying flowering and maturity (Table S2). Amelioration of stress effects however, did help neither growth nor yield. Grain yield attributes decreased in the main shoot and tillers under SF, with effects more visible in Sub1-introgressed cultivars (Tables S1, S2) indicating enhancement of sensitivity. These results conflict with the gain of yield advantage in *Sub1-*introgressed cultivars^36^. Thus, genomic change with *Sub1* allele introgression offers no protection of rice under SF, although environment-specific aberrations could not be ruled out. Singh *et al*.^35^ reported that short-stature cultivars are prone to SF and tolerance depends less on *Sub1-*introgression. Deterioration of yield and yield attributes of rice is common under SF^37–39^ and Swarna and Savitri are no exceptions.

Similar to yield, the *Sub1-*introgressed lines had less tillers under SF, but there was no difference in control (Fig. 2). Singh *et al*.^12^ reported a similar effect of SF in SwarnaSub1. Tiller production was inhibited more in the *Sub1-*introgressed genotype in comparison to the parents because the introgressed-allele restricted stem elongation further under SF. Generally both genetic and environmental factors determine tiller dynamics of rice, but the effects of the former is miniscule compared to the latter^40^. It was shown that high ethylene production was responsible for decimation of late tillers^41^. Presences of water column of 50 cm depth probably restricted carbon assimilate production by photosynthesis and suppressed the development of tiller buds in the present study. Additionally greater water pressure under SF also could have triggered the death of comparatively weaker tillers^42^ because submergence promoted ethylene production^28,35^. Thus, dispensation of weaker tillers or suppression of development of new tillers could be an important strategy for plant survival under SF. It was reported that tillers above 50 cm long survive ≈ 50 cm water depth better than those below this level^43^. Both Swarna and Savitri cultivars exhibited the mechanism of resilience in perturbed situation of SF, by reducing tiller number, yield and yield attributing parameters (Fig. 2, Tables S1 & S2). The introgression of *Sub1* allele to the cultivars became more damaging in place of salvaging the tiller loss. Possibly, incorporation of *Sub1* reduced tillering because it inhibited elongation growth further under SF^12^. Hence, combining *Sub1* locus to short stature semi-dwarf rice provides no security against SF, although it could be useful for taller cultivars.

*Sub1* locus confers tolerance to submergence in rice, yet it also alters some other characteristics. Grain-filling duration extended under SF, although ultimate grain weight was less compared to control due to slow grain-filling rate (Fig. S2; Tables S1 & S2). Slow grain-filling was associated with the delay of whole plant senescence, because flowering and maturity events were delayed under SF (Table S2). In grain maturation period remobilization of assimilates from straw to grain support yield of rice^44,45^. In our study, the gain achieved from extended grain-filling period however, was not enough to bridge the gap in grain yield between control and SF. Slow senescence permitted long duration photosynthesis, but there was no security for greater yield^32^. Grain growth in rice sustains on starch synthesis by consumption of soluble carbohydrates in the endosperm cells. In our study, starch synthesis was impacted by SF leading to accumulation of higher concentration of soluble carbohydrates in the developing spikelets (Figs. 3, 4). This is similar to higher accumulation of sugars in inferior spikelets of rice panicle^17^. The introgression of *Sub1* locus could not provide any protection for amelioration of the stress effects; rather it reduced starch synthesis and very often promoted accumulation of unused soluble carbohydrates in the panicle. This type of effect of SF on grain starch synthesis is in agreement with response of the stress on grain weight and yield of *Sub1-*introgressed cultivars (Fig. S1, Table S2). *Sub1* gene expression is known for its control in suppressing cell wall elongation and carbohydrate metabolism of rice under flooding^46^ and our results are in agreement with these observations.

SUS and AGPase are important enzymes for rice grain-filling^29,47–49^. These enzymes synthesize starch that determines the sink strength of grains^22,50^. The genes encoding the enzymes over-express in superior spikelets compared to the inferior ones in rice panicle^51^. In this study, activities of the enzymes were higher in control compared to SF (Figs. 5, 6), they matched with the concomitant increase of higher grain-filling rate and starch accumulation (Figs. S1, S2, 4). Poor activities of these two enzymes limit grain-filling in under-developed spikelets of rice panicle^48,49,52^ and sugars not used in grain sink for starch synthesis accumulate in the caryopsis^16,53^. SUS activity in our study increased temporally in the first ten days of anthesis and declined thereafter. In comparison, AGPase activity that was slower in the first part of grain-filling period, peaked up after the fall in SUS activity and continued to remain strong for some days in the second part of grain maturation (Figs. 5, 6). SUS is the enzyme that primes entry of phloem-translocated sucrose from the embryonic apoplast into the starch biosynthesis pathway of rice endosperm^22^ leading to cleavage of the disaccharide to UDP-glucose and fructose; the former is phosphorylated to glucose-1-phosphate, which is substrate for AGPase action^21^. High SUS activity in the early part of grain-filling, as observed here, proves its direct linkage to grain sink strength^53^. Because AGPase activity depended on glucose-1-phosphate synthesis, it was low initially on a time scale and gained momentum once SUS action provided its substrate. Consequently greater activity of the enzyme was seen in the later part of grain-filling period. Thus, provision of sucrose not limiting kernel growth, a high SUS-activity at the initial stage and AGPase at the later stage of anthesis is required for proper grain-filling (Figs. 5, 6) and correlation of grain sink strength with activities of enzymes differs on a time scale. Our data suggested that SUS after 5–10 days of anthesis could determine the potential grain yield of rice^53,54^. On the other hand, AGPase activity at mid grain-filling stage could be a potential indicator of grain yield^55,56^. The activities of the enzymes were poorer in *Sub1-*introgressed cultivars compared to their counterparts without *Sub1* under SF. Diminished carbohydrate metabolism caused by the *QTL* under SF could be the factor responsible for this effect^46^.

In rice high ethylene production at anthesis is counter-productive to grain-filling and endosperm starch synthesising enzyme activities^13,14,17,18,24,48,57^. High ethylene production down-plays gene expression encoding these enzymes, which impacts cell division, growth and grain quality^21,58^. At the same time there is a concomitant over-expression of the genes controlling ethylene receptors and signal transducers^59,60^. We observed high concentrations of ethylene under SF (Fig. 7) coincided with poor activities of starch synthesizing enzymes (Figs. 5, 6) and grain-filling rate (Fig. S2). This coincidence strongly favours the concept of ethylene being a causative factor for depression of starch synthesis and grain-filling of rice under SF. Exposition to SF increased ethylene concentration of the panicle of the cultivars irrespective of *Sub-*introgression. The concentration at anthesis was higher in the cultivars with *Sub1* than that of without *Sub1* and endosperm starch synthesis became poorer in the former compared to the latter (Figs. 3, 4). Subjected to SF, all paralogs of ACC-synthase and ACC-oxidase genes in cultivars without *Sub1* over-expressed just before anthesis (Table 1, Fig. S3) and this over-expression could have contributed to the rise of ethylene production. But, the situation was different for the *Sub1-* introgressed cultivars, where most of genes under-expressed, although magnitude of change in expression under SF was almost similar. The response of genes to SF changed dramatically at anthesis, showing consistent down-regulation in expression of the ACC-synthase and ACC-oxidase genes in cultivars without Sub1. In the *Sub1*-introgressed cultivars, expression of most of the paralogs was down-regulated three days before anthesis, supporting the notion that the OTL was effective in reducing ethylene evolution under SF (Table 1). However, some paralogs over-expressed under SF at anthesis and 3 days post-anthesis in SavitriSub1, which might have increased synthesis of ethylene in the panicle later. In particular, one paralog (*OsACS5)* of Swarna-Sub1 and eight paralogs (*OsACS1, OsACS2, OsACS5, OsACS6, OsACO1, OsACO2, OsACO4, OsACO5*) of SavitriSub1 over-expressed at anthesis under SF and they might be responsible for greater synthesis of ethylene. It is known that ERF transcription factor of Sub1 dampens ethylene production that becomes counter-productive to gibberellin action in some rice lines during submergence^27^. In our case, presence of the *QTL* in dampening ethylene synthesis was effective prior to anthesis under SF (Table 1). Typical burst of ethylene evolution at anthesis^57^ under continued submergence might have over-whelmed the suppression effects of Sub1ERF on ethylene synthesis. High ethylene evolution in the Sub1-introgressed cultivars showed profound effects on salient morphological features of the plant like yield, tiller production and stem elongation in our study (Figs. 1, 2). Over-expression of these genes leading to higher evolution of ethylene and decimation of grain-filling in rice has been reported by Panda *et al*.^60^ and the present work corroborates this work. The aberration in *Sub1* behaviour inducing higher evolution of ethylene and concomitant over-expression of the ERF could be consequential to the difference in genotype and manner of flooding of rice cultivars. In our case, *Sub1-*introgression in Swarna did not elicit expression of *ACC-synthase* and *ACC-oxidase* genes similar to that of Savitri. Singh *et al*.^12^ observed that the *QTL* is not effective in semi-dwarf rice cultivars under partial SF; cultivars like Swarna and SwarnaSub1 were more sensitive to long term partial SF than inherently taller cultivars. Earlier we reported greater loss of yield under submergence in SwarnaSub1 compared to traditional cultivar Balidhan^61^ and significant variation among cultivars in stem elongation ability under submergence^31^.These reports denote that resilience of genotypes to SF depends less on *Sub1-*introgression and more so on the genetic make up. This is in conformity with observation of Singh *et al*.^62^ that the physiological basis of tolerance to submergence in rice involves genetic factors in addition to *Sub1* gene. Both Savitri and Swarna are semi-dwarf cultivars and they produced more ethylene for greater elongation of stem under SF irrespective of *Sub1-*introgression. Additionally, *Sub1* might act in a different manner under flash flooding and SF. The *Sub1-*introgressed cultivars of our study were under SF over a long period from the vegetative to grain-filling and their response was not identical to plants encountering other types of flooding. Further, the expression mode of *Sub1* in vegetative tissues in restricting the elongation growth^63^, may not have a similar response in the reproductive tissues. It is concluded that under SF, semi-dwarf *Sub1* cultivars are less suitable for cultivation than the corresponding parental lines. However, this inference reached for the mega semi-dwarf lines used in our study, may not hold true for the cultivars with taller genetic backgrounds that would have different effects under SF. Future studies should investigate the possible differential response on other type of Sub1 cultivars (the ones that have moderate elongation rate under SF and/ or Sub1 cultivar with inherently tall attribute) to see their suitability of planting in the areas that are affected by the combinations of flash flooding and SF stresses.

## Materials and Methods

### Plant material

Two popular rice (*Oryza sativa* L.) cultivars Swarna (Vasista x Mashuri) and Savitri (Pankaj x Jagannath) and their counterpart *Sub1* NILs, SwarnaSub1 and SavitriSub1, developed in ICAR-National Rice Institute, Cuttack, were used. The cultivars are released for shallow rain fed lowland.

### Experimental site

The experiment was conducted in alluvial sandy clay loam soil of the Mahanadi River delta (pH 6.7, organic C 0.89%, total N 0.01%, avialable P 22 kg ha^–1^ and available K 125 kg ha^–1^) at National Rice Research Institute, Cuttack, India (20.5°N, 86°E and 23.5 meters above sea level) during the wet seasons of 2013 and 2014 with three replications under factorial randomized block design. The date of sowing and planting were 21^st^ June and 21^st^ July respectively in the year 2013. For 2014 the respective dates were 9^th^ June and 6^th^ July. Inorganic fertilizers N: P: K was applied at 60:30:30 Kg ha^–1^. Phosphorous as single super phosphate and K as muriate of potash were applied as basal, whereas N as urea was applied in two split doses, 50% after 7 days of transplanting and the rest 50% three days before the imposition of SF. The experiment was conducted in two side by side field tanks (l x b x h: 40 m x 8 m x 0.8 m), in which one was used as control where water depth varied from 2 to 10 cm. The other one was used for SF. 25 days old seedlings were transplanted in the experimental tanks @ single seedling hill^–1^ in a plot (5 m x 3 m). Each tank had twelve plots: hill to hill space was 15 cm and line to line distance was 20 cm. SF was imposed after one month of transplanting by gradual increase of water level @ 10 cm day^–1^. Approximately 50 cm water depth was maintained up to anthesis stage and discontinued thereafter. Water level decreased gradually, yet 5–10 cm standing water stayed in the SF treatment tank at harvest.

### Grain yield attributes

Plants were selected for uniform growth and tagged before the imposition of SF. Yield and yield attributing parameters were measured separately for main shoot and rest of the tillers. Data on plant height and tiller numbers hill^–1^ were collected from ten random hills just before and after the imposition of SF at weekly intervals till maturity. The samples were oven-dried at 65 °C for 3 days before estimation of dry mass.

### Carbohydrate measurement

Panicle of the main shoot was excised at neck node and sampled for estimation of soluble carbohydrates and starch at three day intervals from days 7 to 19 post- anthesis. The harvested panicles were immediately put in an oven at 65 ± 2 °C for estimation of dry weight. Powder of oven dry spikelets of main shoot panicle was boiled in 80% aqueous methanol for 5 min. The extract was collected in a volumetric flask. The residue was boiled again and the second extract was pooled into the flask. The volume of the extract was made up to the mark with distilled water and an aliquot was taken for estimation of soluble carbohydrates^64^. After methanolic extraction the residue was dried, digested with 3% HCl. Glucose released in digestion was used for estimation of starch^53^.

### Enzyme assay

Panicle of the main shoot was excised at three day intervals between days 7 and 19 post-anthesis and snap frozen in liquid nitrogen and stored in a freezer (−80 °C) until the estimation of SUS and AGPase enzyme activities in 500 mg of fresh spikelets.

For SUS activity the plant sample was grinded in 4 ml of ice-cold extraction medium containing Hepes-NaOH buffer (50 mM, pH 7.5), MgCl_2_ (5 mM), Na_2_-EDTA (1 mM), DTT (2.5 mM), bovine serum albumin (1%) and insoluble polyvinyl pyrollidone (0.6%) using mortar and pestle at 4 °C temperature. The extract was centrifuged at 15,000 rpm for 10 min. at 4°c. The supernatant was used as enzyme source for SUS assay^20^.

For AGPase activity, the extract was prepared by grinding 500 mg fresh plant sample in 4 mL extraction medium containing 50 mM Hepes-NaOH buffer (pH 7.5), 5 mM MgCl_2_, 1 mM Na_2_EDTA and 0.5% BSA using mortar and pestle at 4 °C. The extract was centrifuged for 5 min at 12,000 rpm at 4 °C and the supernatant was used as source for the enzyme^20^.

### Ethylene estimation

Panicle of main shoot was cut off for measurement of ethylene three days before to six days after anthesis at three day intervals. The cut end of sample was dipped into water immediately for 10 minutes before sealing it air tight inside a test tube containing a small amount of water^18^. The tube was incubated in darkness for 2 h at room temperature. Headspace gas (1 ml) was drawn in a gas tight syringe and injected into a gas chromatograph (CHEMITO, CERES 800 PLUS, GAS CHROMATOGRAPH) with flame ionization detector. Nitrogen was the carrier gas, and H_2_ and O_2_ were used for flame ionization detector.

### Quantitative real-time PCR and PCR studies

Panicle of the main shoot was excised at the neck node and snap-frozen in liquid nitrogen at three days before to three days after anthesis at three day intervals. The samples were used for quantitative real time PCR to study the transcript level expression of genes involved in ethylene biosynthesis. Total RNA was extracted from all spikelets of frozen (−80 °C) panicle using Chromous Biotech, RNA isolation kit. This RNA pellet was stored at −80 °C. The isolated RNA was converted into c-DNA by using oligo-dT primer according to Chromous biotech (Chromous Biotech Pvt. Ltd., Bangalore, India), RT-PCR teaching kit (c-DNA). This c-DNA was used for quantitative real-time PCR. PCR was standardized to obtain amplification for all the 12 genes for ACC synthase and ACC oxidase enzyme using Rubisco as housekeeping gene. The primer sequences are given in Table S4. qRT-PCR, a real time run was done in ABI-one real time PCR machine. PCR conditions set for gene were *Rubisco*, *OsACS1*, *OsACS2*, *OsACS3*, *OsACS4*, *OsACS5*, *OsACS6*, *OsACO1*, *OsACO2*, *OsACO3*, *OsACO5* and *OsACO7* at 94 °C for 5 min followed by 40 cycles of 94 °C for 5 sec, 60 °C for 10 sec, 72 °C for 10 sec and finally at 72 °C for 5 min. RT-PCR reaction was performed using oligo-dT primer following standard protocol^22,59^. For PCR reaction the first strand c- DNA, forward primer, reverse primer and SYBR green ready mix water of total reaction volume of 50 µl were kept in triplicate and run was done on ABI step-one real time PCR machine.

### Statistical analyses

Statistical analysis for different parameters was done using the CropStat software (International Rice Research Institute, Philippines). Mean values were compared by the least significant difference (LSD, *p < 0.05), wherever the *F*-test was significant.

## Supplementary information

Fig. S1, Fig. S2, Fig. S3, Table S1, Table S2

## References

[1] Das KK, Panda D, Nagaraju M, Sharma SG, Sarkar RK (2004). Antioxidant enzymes and aldehyde releasing capacity of rice cultivars (*Oryza sativa* L.) as determinants of anaerobic seedling establishment capacity. Bulgarian J. Plant Physiol..

[2] Reddy, J.N. *et al*. Improvement of rice germplasm for rainfed lowland of eastern India. *SABRAO J Breed Genet* **41**, Special Supplement, August 2009 (2009)

[3] Singh R (2016). From QTL to variety-harnessing the benefits of QTLs for drought, flood and salt tolerance in mega rice varieties of India through a multi-institutional network. Plant Sci..

[4] Kuanar SR, Ray A, Sethi SK, Chattopadhyay K, Sarkar RK (2017). Physiological basis of stagnant flooding tolerance in rice. Rice Sci..

[5] Neeraja C (2007). A marker-assisted backcross approach for developing submergence-tolerant rice cultivars. Theor. Appl. Genet..

[6] Iftekharuddaula K (2011). Rapid and high-precision marker assisted backcrossing to introgress the SUB1 QTL into BR11, the rainfed lowland rice mega variety of Bangladesh. Euphytica.

[7] Sarkar R, Reddy J, Sharma S, Ismail AM (2006). Physiological basis of submergence tolerance in rice and implications for crop improvement. Curr Sci..

[8] Xu K (2006). Sub1A is an ethylene-response-factor-like gene that confers submergence tolerance to rice. Nature.

[9] Fukao T, Xu K, Ronald PC, Bailey-Serres J (2006). A variable cluster of ethylene response factor–like genes regulates metabolic and developmental acclimation responses to submergence in rice. The Plant Cell.

[10] Panda D, Sarkar RK (2012). Role of non-structural carbohydrate and its catabolism associated with Sub 1 QTL in rice subjected to complete submergence. Exp. Agric..

[11] Sarkar R (2009). Performance of submergence tolerant rice (Oryza sativa) genotypes carrying the Sub1 quantitative trait locus under stressed and non-stressed natural field conditions. Indian J. Agric. Sci..

[12] Singh S, Mackill DJ, Ismail AM (2011). Tolerance of longer-term partial stagnant flooding is independent of the SUB1 locus in rice. Field Crop Res..

[13] Mohapatra PK, Naik PK, Patel R (2000). Ethylene inhibitors improve dry matter partitioning and development of late flowering spikelets on rice panicles. Funct Plant Biol.

[14] Yang J, Zhang J, Wang Z, Liu K, Wang P (2006). Post anthesis development of inferior and superior spikelets in rice in relation to abscissic acid and ethylene. J. Exp. Bot..

[15] Liu K, Ye Y, Tang C, Wang Z, Yang J (2008). Response of ethylene and ACC in rice grains to soil moisture and their relation to grain filling. Frontiers Agric. China.

[16] Mohapatra PK, Patel R, Sahu SK (1993). Time of flowering affects grain quality and spikelet partitioning within rice panicle. Australian J. Plant Physiol..

[17] Panda BB, Kariali E, Panigrahi R, Mohapatra PK (2009). High ethylene production slackens seed filling in compact panicled rice cultivar. Plant Growth Regul..

[18] Naik PK, Mohapatra PK (2000). Ethylene inhibitors enhanced sucrose synthase activity and promoted grain filling of basal rice kernels. Funct Plant Biol..

[19] Yang J, Zhang J (2006). Grain filling of cereals under soil drying. New Phytol..

[20] Mohapatra PK, Sarkar R, Kuanar S (2009). Starch synthesizing enzymes and sink strength of grains of contrasting rice cultivars. Plant Sci..

[21] Panigrahi, R., Kariali, E., Panda, B.B., Lafarge, T. & Mohapatra, P.K. Controlling the trade-off between spikelet number and grain filling; the hierarchy of starch synthesis in spikelets of rice panicle in relation to hormone dynamics. *Funct. Plant Biol*. (accepted) (2019) 10.1071/FP18153 30961785

[22] Panda BB (2015). Compact panicle architecture is detrimental for growth as well as sucrose synthase activity of developing rice kernels. Funct. Plant Biol..

[23] Wang Z, Xu Y, Wang J, Yang J, Zhang J (2012). Polyamine and ethylene interactions in grain filling of superior and inferior spikelets of rice. Plant Growth Regul.

[24] Chen T (2013). Polyamines and ethylene interact in rice grains in response to soil drying during grain filling. J. Exp. Bot..

[25] Yang C, Lu B, Ma B, Chen S-Y, Zhang J-S (2015). Ethylene signaling in rice and Arabidopsis: conserved and diverged aspects. Molecular Plant.

[26] Bailey-Serres J (2010). Submergence tolerant rice: SUB1’s journey from landrace to modern cultivar. Rice.

[27] Fukao T, Yeung E, Bailey-Serres J (2011). The submergence tolerance regulator SUB1A mediates cross talk between submergence and drought tolerance in rice. The Plant Cell.

[28] Hattori Y (2009). the ethylene response factors SNORKEL1 and SNORKEL2 allow rice to adapt to deep water. Nature.

[29] Sarkar RK, Panda D (2009). Distinction and characterisation of submergence tolerant and sensitive rice cultivars, probed by the fluorescence OJIP rise kinetics. Funct Plant Biol.

[30] Bailey-Serres J, Voesenek LA (2010). Life in the balance: a signaling network controlling survival of flooding. Curr. Opin. Plant Biol..

[31] Sarkar RK, Bhattacharjee B (2011). Rice genotypes with SUB1 QTL differ in submergence tolerance, elongation ability during submergence and re-generation growth at re-emergence. Rice.

[32] Panda D, Sarkar RK (2013). Natural leaf senescence: probed by chlorophyll fluorescence, CO2 photosynthetic rate and antioxidant enzyme activities during grain filling in different rice cultivars. Physiol Mol Biol Plants.

[33] Sasaki A (2002). A mutant gibberellin-synthesis gene in rice. Nature.

[34] Spielmeyer W, Ellis MH, Chandler PM (2002). Semidwarf (*sd1*), “green revolution” rice contains a defective gibberellin 20-oxidase gene. PNAS.

[35] Rzewuski G, Sauter M (2008). Ethylene biosynthesis and signaling in rice. Plant Sci.

[36] Singh S, Mackill DJ, Ismail AM (2009). Responses of SUB1 rice introgression lines to submergence in the field: yield and grain quality. Field Crops Res..

[37] Sarkar R, Das S (2003). Yield of rainfed lowland rice with medium water depth under anaerobic direct seeding and transplanting. Trop Sci.

[38] Kato Y, Collard BC, Septiningsih EM, Ismail AM (2014). Physiological analyses of traits associated with tolerance of long-term partial submergence in rice. AoB Plants.

[39] Vergara GV, Nugraha Y, Esguerra MQ, Mackill DJ, Ismail AM (2014). Variation in tolerance of rice to long-term stagnant flooding that submerges most of the shoot will aid in breeding tolerant cultivars. AoB Plants.

[40] Mohapatra Pravat K., Panda Binay B., Kariali Ekamber (2011). Plasticity of Tiller Dynamics in Wild RiceOryza rufipogonGriff.: A Strategy for Resilience in Suboptimal Environments. International Journal of Agronomy.

[41] Kariali E, Mohapatra PK (2007). Hormonal regulation of tiller dynamics in differentially-tillering rice cultivars. Plant Growth Regul..

[42] Reddy B, Ghosh B, Reddy M (1987). Effect of transplanting date and seedling age on stand establishment and grain yield of rice in rainfed lowland (intermediate deep-water) conditions. Exp. Agric..

[43] Singh S, Bhattacharjee D (1988). Effect of water logging on yield and yield attributes in late high yielding rice varieties. Oryza.

[44] Yang J, Zhang J (2010). Crop management techniques to enhance harvest index in rice. J. Exp. Bot..

[45] Zhang H, Chen T, Wang Z, Yang J, Zhang J (2010). Involvement of cytokinins in the grain filling of rice under alternate wetting and drying irrigation. J. Exp. Bot..

[46] Nugraha Y, Vergara GV, Mackill DJ, Ismail AB (2013). Response of SUB1 introgression lines of rice to various flooding conditions. Indonesian J. Agric. Sci..

[47] Kato T (1995). Change of sucrose synthase activity in developing endosperm of rice cultivars. Crop Sci..

[48] Tang T, Xie H, Wang Y, Lü B, Liang J (2009). The effect of sucrose and abscisic acid interaction on sucrose synthase and its relationship to grain filling of rice (*Oryza sativa* L.). J. Exp. Bot..

[49] Zhu G, Ye N, Yang J, Peng X, Zhang J (2011). Regulation of expression of starch synthesis genes by ethylene and ABA in relation to the development of rice inferior and superior spikelets. J. Exp. Bot..

[50] Liang J, Zhang J, Cao X (2001). Grain sink strength may be related to the poor grain filling of indica‐japonica rice (*Oryza sativa*) hybrids. Physiol. Plant..

[51] You C (2016). Effect of removing superior spikelets on grain filling of inferior spikelets in rice. Front. Plant Sc..

[52] Zhang H (2012). Post-anthesis alternate wetting and moderate soil drying enhances activities of key enzymes in sucrose-to-starch conversion in inferior spikelets of rice. J. Exp. Bot..

[53] Patel R, Mohapatra PK (1996). Assimilate partitioning within floret components of contrasting rice spikelets producing qualitatively different types of grains. Australian J. Plant Physiol..

[54] Counce PA, Gravois KA (2006). Sucrose synthase activity as a potential indicator of high rice grain yield. Crop Sci..

[55] Kato T, Shinmura D, Taniguchi A (2007). Activities of enzymes for sucrose-starch conversion in developing endosperm of rice and their association with grain filling in extra-heavy panicle types. Plant Prod. Sci..

[56] Kato T, Taniguchi A, Horibata A (2010). Effects of the alleles at OsAPGS2 and OsSUT1 on the grain filling of extra heavy panicle type of rice. Crop Sci..

[57] Kuanar SR, Panigrahi R, Kariali E, Mohapatra PK (2010). Apoplasmic assimilates and grain growth of contrasting rice cultivars differing in grain dry mass and size. Plant Growth Regul.

[58] Mohapatra PK, Panigrahi R, Turner NC (2011). Physiology of spikelet development in rice: is manipulation of apical dominance crucial for grain yield. Adv. Agron..

[59] Sekhar S (2015). Spikelet-specific variation in ethylene production and constitutive expression of ethylene receptors and signal transducers during grain filling of compact- and lax-panicle rice (*Oryza sativa*) cultivars. J. Plant Physiol..

[60] Panda BB (2016). 1-MCP treatment enhanced expression of genes controlling endosperm cell division and starch biosynthesis for improvement of grain filling in a dense-panicle rice cultivar. Plant Sci..

[61] Ray Anuprita, Panda Debabrata, Sarkar Ramani Kumar (2016). Can rice cultivar with submergence tolerant quantitative trait locus (SUB1) manage submergence stress better during reproductive stage?. Archives of Agronomy and Soil Science.

[62] Singh S, Mackill D, Ismail AB (2014). Physiological basis of tolerance to complete submergence in rice involves genetic factors in addition to the Sub1 gene. AoB Plants.

[63] **X**u, K., Ismail, A. M. & Ronald, P. Flood tolerance mediated by the rice Sub1A transcription factor. *In* Jenks, M. A. & Hasegawa, P. M. E., eds, Plant abiotic stress. Second edition. John Wiley and Sons, New York, pp 1–13 (2014).

[64] Pattanaik PK, Mohapatra PK (1988). Role of assimilates and phosphates in the control of internode elongation in tall and dwarf indica rice varieties. J. Exp. Bot..

