# Supplementary material for: Introgression of Sub1 (SUB1) QTL in mega rice cultivars increases ethylene production to the detriment of grain- filling under stagnant flooding
Source: Sci Rep. 2019 Dec 6;9:18567. doi: 10.1038/s41598-019-54908-2 (PMC6898156; doi:10.1038/s41598-019-54908-2)
Supplement: Supplementary file 1 — Fig. S1, Fig. S2, Fig. S3, Table S1, Table S2 [file 41598_2019_54908_MOESM1_ESM.pdf]

Introgression of *Sub1 (SUB1) QTL* in mega rice cultivars increases ethylene production to the detriment of grain- filling under stagnant flooding.

Sandhya Rani Kuanar[a, c] , Kutubuddin Ali Molla[a], Krishnendu Chattopadhyay[a], Ramani Kumar Sarkar[a] , Pravat Kumar Mohapatra[b *]

[a]ICAR-National Rice Research Institute, Cuttack, Odisha 753 006, India.

[b]School of Life Science, Sambalpur University, Jyoti vihar, Sambalpur 768019, India.

[c] Present address- Anchal College, Padampur, 768036, India

*Corresponding author

Email IDs:

Sandhya Rani Kuanar: sandhya_kuanar@rediffmail.com

Kutubuddin Ali Molla: Kutubuddin.Molla@icar.gov.in

Krishendu Chattopadhyay: krishnenducrri@gmail.com

Ramani Kumar Sarkar: rksarkarcrri@gmail.com

Pravat Kumar Mohapatra: pravat1948@rediffmail.com Mobile -91-9937103747

Legend for figures

Fig. S1 Effect of SF on grain yield of main shoot. Refer Fig.1 for symbols.

Fig.S2 Effect of SF on grain-filling rate of main shoot. Refer Fig.1 for symbols.

Fig. S3: Relative mRNA level of various isoforms of ethylene biosynthetic genes ACC-synthase and ACC-oxidase in panicle of Swarna and Swarna-Sub1 (A) and Savitri and Savitri-Sub1 (B) under SF during 2014 compared to control on -3,0 and 3 days from anthesis. The horizontal bars indicate mean ± standard deviation (n = 3). Absence of bar at any point indicates that the value was too small for representation.

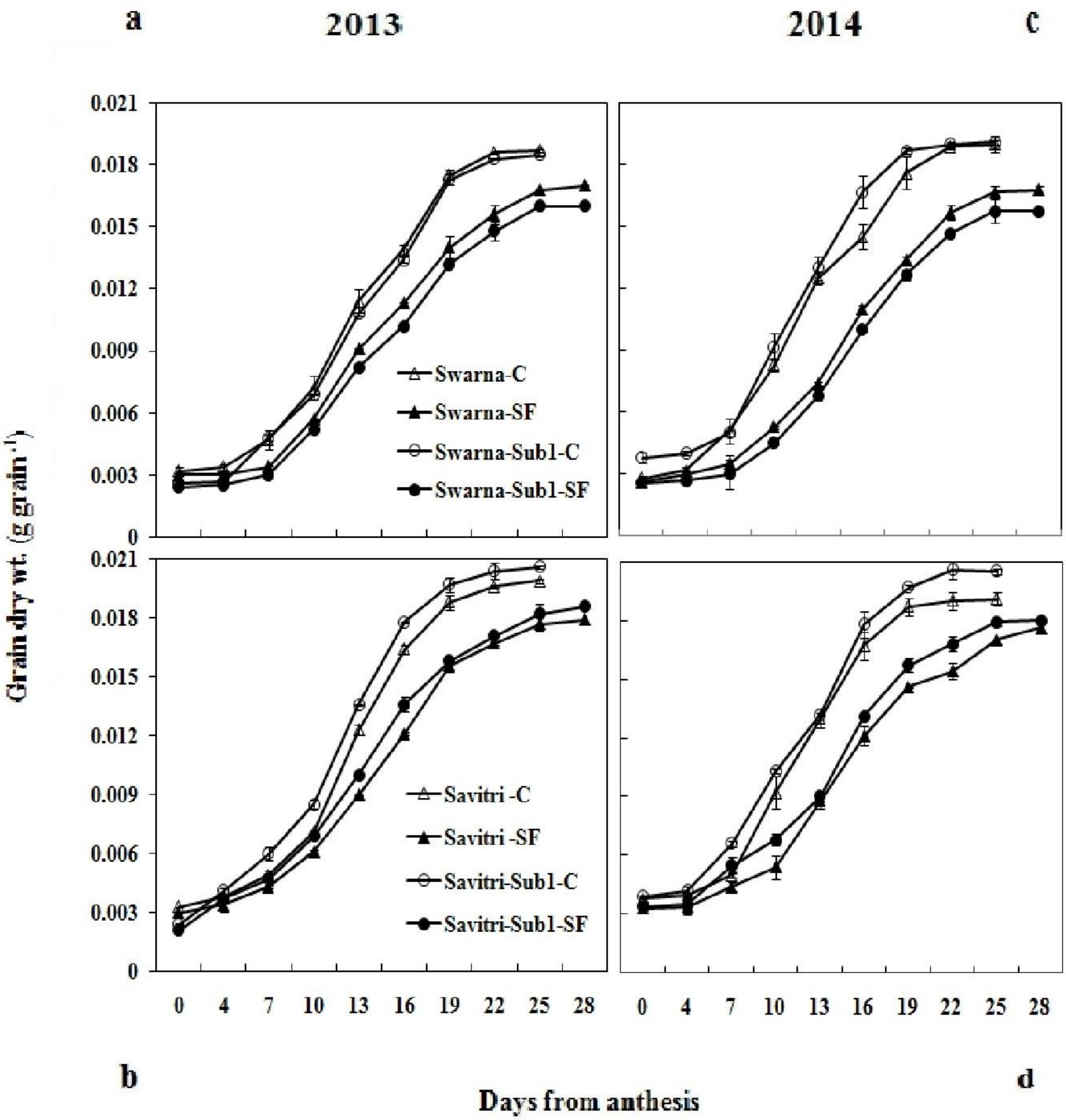

a
2013
2014
c
0.021
0.018
0.015
0.012
0.009
0.006
0.003
0
Swarna-C
Swarna-SF
Swarna-Sub1-C
Swarna-Sub1-SF
Savitri -C
Savitri -SF
Savitri-Sub1-C
Savitri-Sub1-SF
Grain dry wt. (g grain⁻¹)
0 4 7 10 13 16 19 22 25 28
b
d
Days from anthesis

Fig. S1

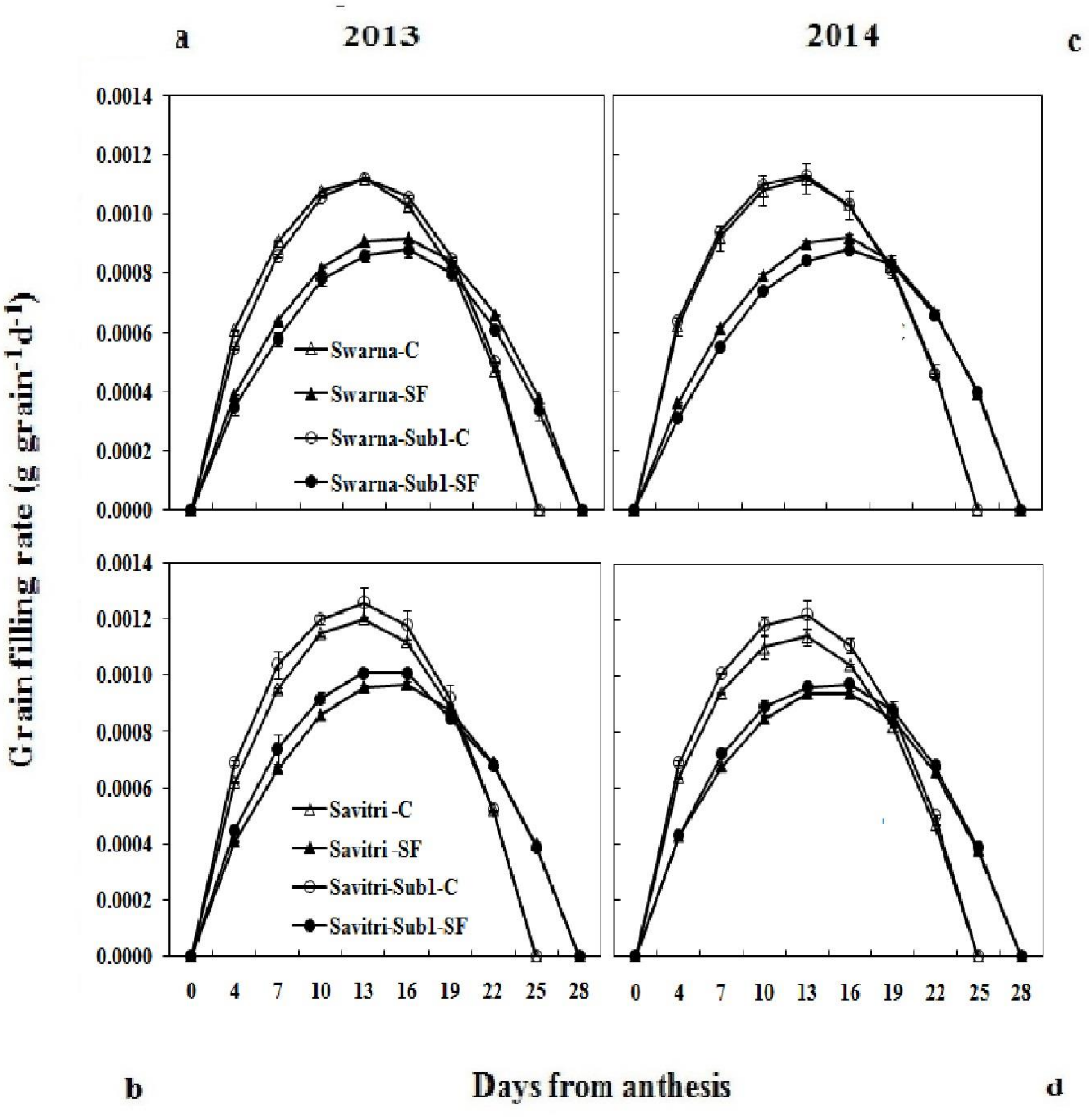
a
2013
2014
c
Grain filling rate (g grain-1 d-1)
Swarna-C
Swarna-SF
Swarna-Sub1-C
Swarna-Sub1-SF
Savitri -C
Savitri -SF
Savitri-Sub1-C
Savitri-Sub1-SF
0.0014
0.0012
0.0010
0.0008
0.0006
0.0004
0.0002
0.0000
0 4 7 10 13 16 19 22 25 28
b
Days from anthesis
d

Fig.S2

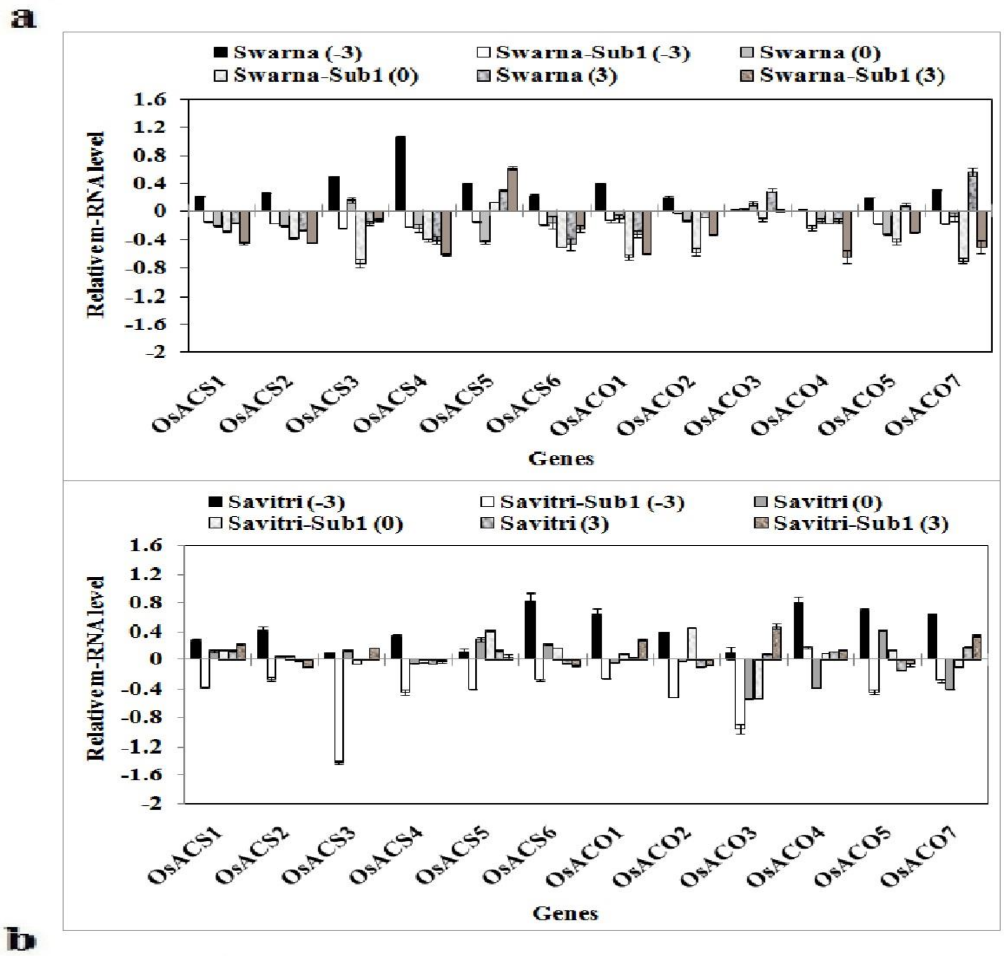
a
Swarna (-3)
Swarna-Sub1 (-3)
Swarna (0)
Swarna-Sub1 (0)
Swarna (3)
Swarna-Sub1 (3)
Relative m-RNA level
1.6
1.2
0.8
0.4
0
-0.4
-0.8
-1.2
-1.6
-2
OsACS1
OsACS2
OsACS3
OsACS4
OsACS5
OsACS6
OsACO1
OsACO2
OsACO3
OsACO4
OsACO5
OsACO7
Genes
Savitri (-3)
Savitri-Sub1 (-3)
Savitri (0)
Savitri-Sub1 (0)
Savitri (3)
Savitri-Sub1 (3)
Relative m-RNA level
1.6
1.2
0.8
0.4
0
-0.4
-0.8
-1.2
-1.6
-2
OsACS1
OsACS2
OsACS3
OsACS4
OsACS5
OsACS6
OsACO1
OsACO2
OsACO3
OsACO4
OsACO5
OsACO7
Genes
b

Fig. S3

Table S1

A.

Genotype	Treatment	Panicle weight (g)	Grain weight (g panicle^{-1})	Filled grain (%)	Panicle number (m^{-2})	Grain weight (g m^{-2})	Straw weight (g m^{-2})
Swarna	C	2.78±0.05	2.56±0.06	83±2	246±10	635±10.8	666±14.6
	SF	2.71±0.07	2.40±0.09	79±11	178±18	464±15.2	601±21.3
Swarna-Sub1	C	2.92±0.03	2.61±0.05	85±3	240±08	626±11.7	671±14.3
	SF	1.57±0.09	1.21±0.10	58±14	123±16	187±16.2	495±18.1
Savitri	C	2.81±0.04	2.52±0.07	75±4	264±09	654±9.3	834±13.9
	SF	2.64±0.05	2.24±0.11	68±9	186±17	443±18.6	685±24.0
Savitri-Sub1	C	3.08±0.07	2.58±0.06	74±4	255±09	657±13.2	824±12.8
	SF	2.80±0.10	2.28±0.12	64±15	132±15	325±19.9	658±20.1
$LSD_{*p<0.05}$	Variety (V)	0.48	0.45	6	36	70	78
	Treatment (T)	0.34	0.31	5	26	50	55
	V X T	0.68	0.63	9	52	100	110

B.

Genotype	Treatment	Panicle weight (g)	Grain weight (g panicle^{-1})	Filled grain (%)	Panicle number (m^{-2})	Grain weight (g m^{-2})	Straw weight (g m^{-2})
Swarna	C	3.18±0.04	2.76±0.03	94±3	244±08	673±12.5	721±14.5
	SF	3.11±0.06	2.68±0.05	81±8	182±13	488±14.9	518±20.8
Swarna-Sub1	C	3.15±0.03	2.66±0.04	93±2	249±09	662±11.8	703±16.0
	SF	2.68±0.05	2.18±0.07	76±10	118±10	264±19.5	445±25.0
Savitri	C	2.88±0.03	2.52±0.03	79±5	270±12	680±10.6	926±15.4
	SF	2.74±0.05	2.27±0.08	66±12	223±14	468±16.7	723±24.7
Savitri-Sub1	C	2.97±0.06	2.59±0.05	82±5	265±11	686±10.2	966±12.8
	SF	2.82±0.08	2.25±0.07	65±13	165±13	363±18.0	717±23.5
$LSD_{*p<0.05}$	Variety (V)	0.33	0.37	5	21	55	104
	Treatment (T)	0.24	0.26	3	15	39	73
	V X T	0.47	0.53	7	30	78	147

The effect of SF on yield and yield attributes of primary and secondary tillers (except main shoot) of rice cultivars Swarna and Savitri with and without *Sub1* during wet season of 2013 (A) and 2014 (B). To calculate grain weight and straw weight m^{-2} main shoot was also included. The data represented as mean ± standard deviation (n = 3) for 2013 and (n = 6) for 2014; [C-Control, SF- Stagnant flooding]

Table S2

A.

Genotype	Treatment	Flowering date	Maturity date	Plant height (cm)	Panicle weight (g)	Grain weight (g) panicle^{-1}	Filled grain no. Panicle^{-1}	Unfilled grain no. Panicle^{-1}	Filled grain (%)
Swarna	C	Oct'16	Nov'16	115±0.8	3.50±0.04	3.27±0.04	163±9	20±2	89±1
	SF	Oct'19	Nov'22	133±1.5	3.35±0.06	3.10±0.05	173±8	25±2	87±2
Swarna-Sub1	C	Oct'20	Nov'22	111±1.0	3.57±0.03	3.33±0.05	184±6	18±2	91±1
	SF	Oct'25	Dec'3	115±1.4	3.32±0.07	3.04±0.07	172±2	40±4	81±1
Savitri	C	Nov'10	Dec'12	116±0.9	3.74±0.03	3.41±0.03	170±5	32±3	85±2
	SF	Nov'13	Dec'17	127±1.8	3.64±0.04	3.19±0.06	154±7	40±5	79±4
Savitri-Sub1	C	Oct'30	Nov'30	114±0.7	4.03±0.05	3.90±0.05	164±4	31±3	84±1
	SF	Nov'3	Dec'07	118±1.4	3.84±0.06	3.44±0.04	180±3	56±2	75±1
		LSD*p<0.05	Variety (V)	4	0.14	0.18	8	5	2.9
			Treatment (T)	3	0.10	0.12	6	4	2.0
			V X T	5	0.19	0.25	11	7	4.0
B.									
Swarna	C	Oct'1	Oct'31	112±0.9	3.85±0.04	3.71±0.04	182±4	13±4	93±2
	SF	Oct'7	Nov'10	126±1.3	3.68±0.08	3.50±0.05	180±7	27±3	87±4
Swarna-Sub1	C	Oct'4	Nov'4	109±0.8	3.90±0.05	3.62±0.03	186±6	17±3	91±2
	SF	Oct'12	Nov'17	112±1.4	3.58±0.09	3.23±0.07	183±4	39±5	83±2
Savitri	C	Oct'25	Nov'26	115±1.1	3.95±0.03	3.50±0.02	169±7	30±4	85±2
	SF	Oct'29	Dec'1	129±1.4	3.77±0.07	3.26±0.06	151±9	41±4	79±2
Savitri-Sub1	C	Oct'20	Nov'20	117±1.0	4.43±0.03	4.05±0.04	174±1	31±4	85±2
	SF	Oct'25	Nov'27	120±1.6	4.22±0.04	3.72±0.06	172±8	65±7	73±4
		LSD*p<0.05	Variety (V)	2	0.15	0.16	7	5	2.2
			Treatment (T)	3	0.10	0.12	5	4	2.0
			V X T	3	0.21	0.22	10	7	3.1

. The effect of SF on yield and yield attributes of main shoot of rice plant with and without *SUB1* during wet season of 2013 (A) and 2014 (B). The value represented as mean ± standard deviation (n = 3) for 2013 and (n = 6) for 2014; Oct, October; Nov, November; Dec, December; C, Control; SF, Stagnant flooding.
